# Supplementary material for: MetaRibo-Seq measures translation in microbiomes
Source: Nat Commun. 2020 Jun 29;11:3268. doi: 10.1038/s41467-020-17081-z (PMC7324362; doi:10.1038/s41467-020-17081-z)
Supplement: Supplementary file 10 — Supplementary Data 7 [file 41467_2020_17081_MOESM10_ESM.zip › File2/Confidence_High_Taxonomy/436070_out.krona.html]

Javascript must be enabled to view this page.

members
magnitude
magnitudeUnassigned
count
unassigned
taxon
rank

436070\_out

3

superkingdom
3
2

3
1239
phylum

186801
3
class

186802
3
order

family
31979
3

3
1485
genus

1262827
1
species

SRS024132\_contig\_number\_39339

1
1896986
species

SRS150029\_contig\_number\_14602

1262788
1
species

SRS013476\_contig\_number\_contig-100\_1917.218125
